# Modeling CADASIL vascular pathologies with patient-derived induced pluripotent stem cells

**DOI:** 10.1007/s13238-019-0608-1

**Published:** 2019-02-18

**Authors:** Chen Ling, Zunpeng Liu, Moshi Song, Weiqi Zhang, Si Wang, Xiaoqian Liu, Shuai Ma, Shuhui Sun, Lina Fu, Qun Chu, Juan Carlos Izpisua Belmonte, Zhaoxia Wang, Jing Qu, Yun Yuan, Guang-Hui Liu

**Affiliations:** 1 Advanced Innovation Center for Human Brain Protection, National Clinical Research Center for Geriatric Disorders, Xuanwu Hospital Capital Medical University, Beijing, 100053 China; 2 Department of Neurology, Peking University First Hospital, Beijing, 100034 China; 3 National Laboratory of Biomacromolecules, CAS Center for Excellence in Biomacromolecules, Institute of Biophysics, Chinese Academy of Sciences, Beijing, 100101 China; 4 State Key Laboratory of Stem Cell and Reproductive Biology, Institute of Zoology, Chinese Academy of Sciences, Beijing, 100101 China; 5 State Key Laboratory of Membrane Biology, Institute of Zoology, Chinese Academy of Sciences, Beijing, 100101 China; 6 University of Chinese Academy of Sciences, Beijing, 100049 China; 7 Gene Expression Laboratory, Salk Institute for Biological Studies, 10010 North Torrey Pines Road, La Jolla, CA 92037 USA; 8 Institute for Stem cell and Regeneration, CAS, Beijing, 100101 China; 9 Key Laboratory of Regenerative Medicine of Ministry of Education, Institute of Aging and Regenerative Medicine, Jinan University, Guangzhou, 510632 China; 10 Beijing Institute for Brain Disorders, Capital Medical University, Beijing, 100069 China

**Keywords:** CADASIL, iPSC, NOTCH, NF-κB, vascular smooth muscle

## Abstract

Cerebral autosomal dominant arteriopathy with subcortical infarcts and leukoencephalopathy (CADASIL) is a rare hereditary cerebrovascular disease caused by a *NOTCH3* mutation. However, the underlying cellular and molecular mechanisms remain unidentified. Here, we generated non-integrative induced pluripotent stem cells (iPSCs) from fibroblasts of a CADASIL patient harboring a heterozygous *NOTCH3* mutation (c.3226C>T, p.R1076C). Vascular smooth muscle cells (VSMCs) differentiated from CADASIL-specific iPSCs showed gene expression changes associated with disease phenotypes, including activation of the NOTCH and NF-κB signaling pathway, cytoskeleton disorganization, and excessive cell proliferation. In comparison, these abnormalities were not observed in vascular endothelial cells (VECs) derived from the patient’s iPSCs. Importantly, the abnormal upregulation of NF-κB target genes in CADASIL VSMCs was diminished by a NOTCH pathway inhibitor, providing a potential therapeutic strategy for CADASIL. Overall, using this iPSC-based disease model, our study identified clues for studying the pathogenic mechanisms of CADASIL and developing treatment strategies for this disease.

**Electronic supplementary material:**

The online version of this article (10.1007/s13238-019-0608-1) contains supplementary material, which is available to authorized users.

## Introduction

Cerebral autosomal dominant arteriopathy with subcortical infarcts and leukoencephalopathy (CADASIL), a hereditary cerebrovascular disease caused by a *NOTCH3* gene mutation (Joutel et al., 1996; Goate and Morris, 1997; Rutten et al., 2014), has the clinical manifestations of recurrent ischemic stroke, progressive cognitive decline and mental disorders (Wang et al., 2011; Di Donato et al., 2017; Fang et al., 2017). The average age at onset for CADASIL is approximately 40 years, which is younger than that of many other non-hereditary cerebrovascular diseases (Herve and Chabriat, 2010; Wang, 2018). Due to early onset and the lack of effective therapy, CADASIL patients face a serious risk of poor quality of life and eventually death.

Blood vessel walls are composed of three layers: the tunica intima, tunica media and tunica adventitia. The tunica intima mainly consists of vascular endothelial cells (VECs) and connective tissues. The structure of the tunica media varies in different vessels, with abundant parallel elastic fibers and vascular smooth muscle cells (VSMCs) in large and medium arteries but mainly VSMCs in small arteries and veins (Swift and Weinstein, 2009; Krings et al., 2011). NOTCH3 is predominantly expressed in the vascular system and is particularly important for the maturation of VSMCs (Villa et al., 2001; Domenga et al., 2004; Liu et al., 2010; Jin et al., 2014; Granata et al., 2015; Gatti et al., 2018). Consistent with the tissue localization and function of NOTCH3, CADASIL mainly affects VSMCs in the tunica media. The specific pathological feature of CADASIL is the deposition of granular osmiophilic material (GOM) on the basement membrane of VSMCs, which is accompanied by prominent thickening of vessel walls due to the deposition of various extracellular matrix proteins (Tikka et al., 2009; Dong et al., 2012; Monet-Lepretre et al., 2013; Zhang et al., 2015b; Capone et al., 2016). Abnormalities in proliferation ability, mitochondrial function and cytoskeleton structure have also been identified in VSMCs from CADASIL patients and mice (Domenga et al., 2004; Tikka et al., 2012; Viitanen et al., 2013; Panahi et al., 2018). Despite these prior studies, detailed phenotypic profiles of VSMCs and other types of cells in CADASIL patients, such as VECs, and the underlying mechanism of CADASIL remain elusive.

Study of the pathogenesis of CADASIL is limited, largely due to a lack of appropriate experimental models. CADASIL mouse models have been used to study CADASIL-specific GOM deposits and vascular dysfunction (Shibata et al., 2004; Lacombe et al., 2005; Joutel et al., 2010). However, such mice are mostly transgenic animals that overexpress mutant human or rodent NOTCH3 and thus have different genotypes than CADASIL patients (Joutel, 2011). Immortalized primary VSMCs derived from CADASIL patients have transformation-related artifacts and are difficult to obtain due to the rarity of CADASIL. Thus, a model that not only faithfully represents disease-associated defects but also is applicable for patients is urgently needed. In recent years, the development of somatic cell reprogramming and *in vitro* directed differentiation techniques have provided effective approaches for modeling disease-specific phenotypes, conducting pathogenesis research and performing drug screening (Li et al., 2011; Liu et al., 2011a, b, 2012, 2014; Fu et al., 2016; Li and Izpisua Belmonte, 2016; Wang et al., 2017).

Here, we generated a non-integrative iPSC-based disease model for CADASIL and obtained CADASIL-specific VSMCs and VECs. In CADASIL VSMCs, phenotype-associated aberrant transcripts and disease-associated cellular dysfunction, including NOTCH and NF-κB pathway activation, cytoskeleton disorganization, and elevated cell proliferation, were identified. Treatment with a NOTCH pathway inhibitor alleviated the upregulation of NF-κB target genes in CADASIL VSMCs, suggesting a potential pharmacological intervention strategy for CADASIL. Overall, we established an iPSC-based disease model for CADASIL and thereby provided valuable clues for pathogenic analysis and therapeutic strategy development.

## Results

### Generation of CADASIL-specific non-integrative iPSCs

**Figure 1:**
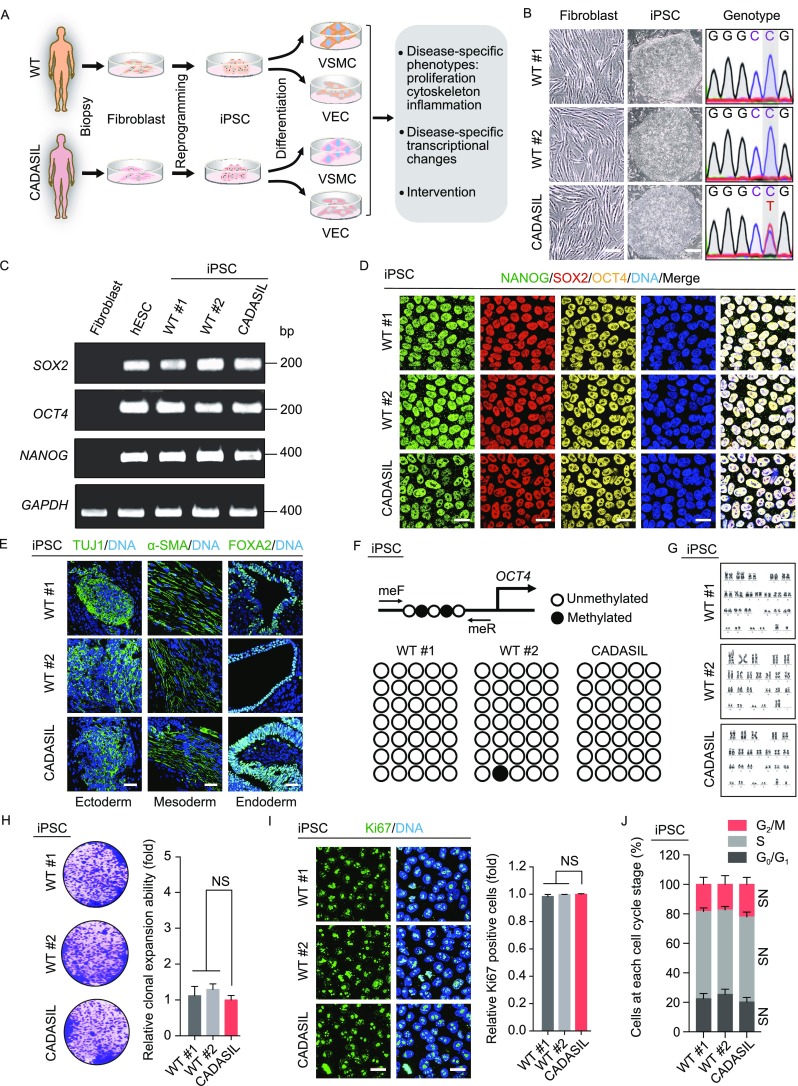
Generation and characterization of WT iPSCs and CADASIL iPSCs. (A) Schematic procedures for establishing iPSC-based CADASIL disease model. Fibroblasts obtained from one CADASIL patient and two healthy controls were reprogrammed into iPSCs. The iPSCs were then differentiated to generate VSMCs and VECs. Changes in disease-associated transcriptional profiling and cellular phenotypes were analyzed. (B) Confirmation of the heterozygous mutation of *NOTCH3* (c.3226C>T, p.R1076C) in CADASIL iPSCs by DNA sequencing (right). Phase-contrast images of fibroblasts (left) and fibroblast-derived iPSCs (middle). Scale bar of fibroblasts, 50 μm; Scale bar of iPSCs, 100 μm. (C) RT-PCR of pluripotency markers, *SOX2*, *OCT4*, and *NANOG*. Human ESCs (hESCs) were used as positive controls and human fibroblasts as negative controls. (D) Immunofluorescence staining of pluripotency markers, NANOG, SOX2, and OCT4. Nuclei were stained with Hoechst 33342. Scale bar, 25 μm. (E) Immunofluorescence staining of TUJ1 (ectoderm), α-SMA (mesoderm), and FOXA2 (endoderm) in teratomas derived from WT and CADASIL iPSCs. Nuclei were stained with Hoechst 33342. Scale bar, 50 μm. (F) DNA methylation analysis of the *OCT4* promoter in WT and CADASIL iPSCs. Open and closed circles indicate unmethylated and methylated CpG dinucleotides, respectively (*n* = 7). (G) Karyotyping analysis of WT and CADASIL iPSCs. (H) Clonal expansion analysis of WT and CADASIL iPSCs. Representative images of crystal violet staining are shown to the left. The statistical analyses of relative clonal expansion abilities are shown to the right (CADASIL was taken as reference). Data are presented as mean ± SD, *n* = 3. NS, not significant. (I) Immunofluorescence staining of Ki67 in WT and CADASIL iPSCs. Nuclei were stained with Hoechst 33342. Scale bar, 25 μm. The relative percentages of Ki67-positive cells are shown to the right (CADASIL was taken as reference). Data are presented as mean ± SD, *n* = 3. NS, not significant. (J) Cell cycle analysis of WT and CADASIL iPSCs. Data are presented as mean ± SD, *n* = 3. NS, not significant

To model CADASIL, we obtained human primary fibroblasts from one CADASIL patient and two healthy controls (WTs) and generated patient-specific iPSCs and WT iPSCs via ectopic expression of *OCT4*, *SOX2*, *KLF4*, *MYCL*, *LIN28* and simultaneous knockdown of *P53* (Li et al., 2011; Liu et al., 2011a, 2014; Okita et al., 2011; Wang et al., 2017) (Fig. 1A). Heterozygous mutations of the *NOTCH3* gene (c.3226C>T, p.R1076C) in CADASIL fibroblasts and iPSCs were verified via genomic PCR and sequencing (Fig. 1B). No significant difference in reprogramming efficiency was observed between WT and CADASIL fibroblasts, and no integrated foreign genes were detected in any of the three iPSC lines (Fig. S1A and S1B). The generated iPSCs exhibited comparable levels of the pluripotency markers OCT4, SOX2 and NANOG (Fig. 1C and 1D); developed teratomas consisting of three germ layers *in vivo* (Fig. 1E); maintained hypomethylated CpG islands in the promoter of *OCT4* (Fig. 1F); and exhibited normal karyotypes (Fig. 1G). Clonal expansion, Ki67 immunofluorescence staining, and cell cycle analysis indicated that all three iPSC lines had similar proliferative abilities (Fig. 1H–J). Taken together, CADASIL-specific iPSCs were generated with normal pluripotency and proliferation abilities.

### Transcriptional profile changes in CADASIL VSMCs

**Figure 2:**
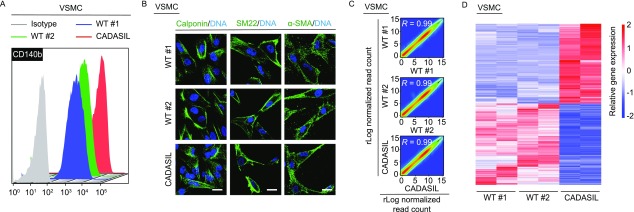

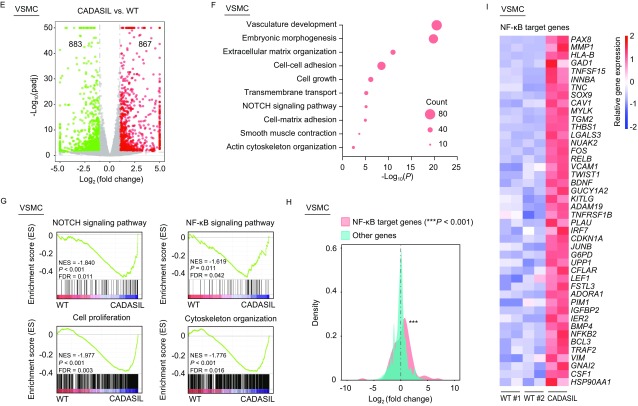
Transcriptional profiling changes in CADASIL VSMCs. (A) Flow cytometry analysis of VSMC-specific marker CD140b in WT and CADASIL VSMCs. (B) Immunofluorescence staining of VSMC-specific markers, Calponin, SM22 and α-SMA. Nuclei were stained with Hoechst 33342. Scale bar, 25 μm. (C) Scatter plots showing the correlation between replicates of WT and CADASIL VSMCs. (D) Heatmap illustrating differentially expressed genes in WT and CADASIL VSMCs. (E) Volcano plot showing the number of upregulated (red dot) and downregulated (green dot) genes in CADASIL VSMCs. (F) GO enrichment analysis of upregulated genes in CADASIL VSMCs. (G) Gene set enrichment analysis (GSEA) plots showing representative GO-BP terms enriched in CADASIL VSMCs. (H) Density plot showing Log_2_(fold change) of mRNA expression levels between WT and CADASIL VSMCs for NF-κB target genes. A rightward shift (***P < 0.001) indicates increased frequency of genes upregulated in CADASIL VSMCs. (I) Heatmap showing upregulated NF-κB target genes in CADASIL VSMCs

Previous studies have demonstrated that CADASIL mainly affects VSMCs (Okeda et al., 2002; Miao et al., 2004, 2006). To investigate functional defects in CADASIL-specific VSMCs, we differentiated CADASIL and WT iPSCs into VSMCs. The derived VSMCs expressed comparable levels of the VSMC-specific markers CD140b, calponin, SM22 and α-SMA (Fig. 2A and 2B). RNA sequencing was performed, and the high correlation coefficients between replicates confirmed high reproducibility (Fig. 2C). There were 867 upregulated genes and 883 downregulated genes in CADASIL VSMCs compared with WT VSMCs (|log_2_(fold change)| > 1, adjusted *P* value (padj) < 0.05) (Fig. 2D and 2E). Gene ontology biological processes (GO-BP) analysis revealed that the upregulated genes in CADASIL VSMCs were enriched in gene terms associated with vasculature development, extracellular structure organization, cell growth, NOTCH signaling, and actin cytoskeleton organization (Fig. 2F). Consistently, gene set enrichment analysis (GSEA) data revealed that compared with control cells, CADASIL VSMCs were enriched in genes associated with various GO terms, including “NOTCH signaling pathway”, “NF-κB signaling pathway”, “cell proliferation”, and “cytoskeleton organization” (Fig. 2G). In contrast to previous studies, which have never reported CADASIL-related activation of the NF-κB signaling pathway, in this study, we noticed that multiple NF-κB target genes were upregulated in CADASIL VSMCs (Fig. 2H and 2I). Certain upregulated genes were closely related to vascular dysfunction and inflammatory response, such as *THBS1*, *MMP1*, *ADAM19* and *TNFSF15* (Bonnefoy et al., 2008; Edwards et al., 2008; Kim et al., 2008; Bin et al., 2009; Penn et al., 2014). Upregulated genes in CADASIL VSMCs were further verified by RT-qPCR (Fig. S1C). GO-BP analysis and RT-qPCR were also used to verify downregulated genes in CADASIL VSMCs (Fig. S1D and S1E). Overall, we generated CADASIL-specific VSMCs and noticed transcriptional profiling changes related to the NOTCH signaling pathway, the NF-κB signaling pathway, cell proliferation, and cytoskeleton disorganization.

### Activation of NF-κB in CADASIL VSMCs was related to NOTCH pathway upregulation

**Figure 3:**
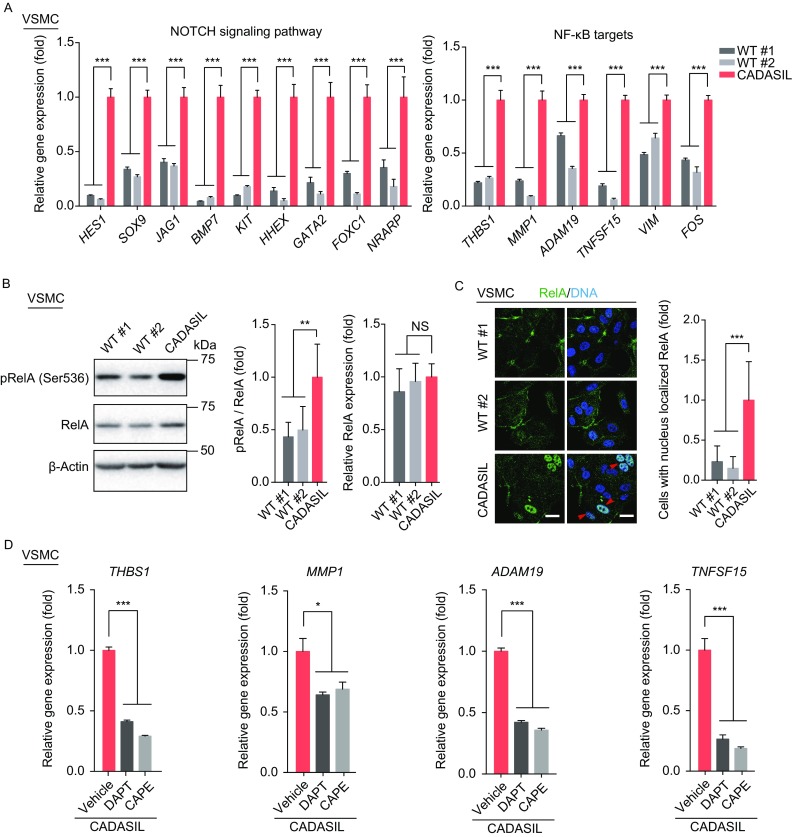
Activation of NF-κB in CADASIL VSMCs was related to NOTCH pathway upregulation. (A) Verification of upregulated NOTCH pathway genes and NF-κB target genes in CADASIL VSMCs by RT-qPCR. CADASIL was taken as reference. Data are presented as mean ± SEM, *n* = 4. ***P < 0.001. (B) Western blot analysis of NF-κB P65 (RelA) and phosphorylated RelA (Ser536) expression levels in WT and CADASIL VSMCs. β-Actin was used as the loading control. Data are presented as mean ± SD, *n* = 5. NS, not significant. **P < 0.01. (C) Immunofluorescence staining of NF-κB P65 (RelA) in WT and CADASIL VSMCs. Nuclei were stained with Hoechst 33342. Scale bar, 25 μm. The relative percentages of cells with nucleus localized RelA are shown to the right (CADASIL was taken as reference). Data are presented as mean ± SD, *n* = 3. ***P < 0.001. (D) RT-qPCR analysis of NF-κB target genes in CADASIL VSMCs. CADASIL VSMCs were treated with 20 μmol/L DAPT (GSI-IX) (Selleck, S2215) and 50 μmol/L Caffeic Acid Phenethyl Ester (CAPE) (Selleck, S7414) for 18 hours respectively. Vehicle was taken as reference. Data are presented as mean ± SEM, *n* = 4. *P < 0.05, ***P < 0.001

Upregulated genes associated with the NOTCH pathway and NF-κB targets identified by RNA-seq in CADASIL VSMCs were further verified by RT-qPCR (Fig. 3A). To investigate whether the NF-κB pathway is activated in these cells, we examined the activation state of NF-κB P65 (RelA), a subunit of the NF-κB heterodimer. We found increased phosphorylation of RelA and an increased proportion of nucleus-localized RelA in CADASIL VSMCs (Fig. 3B and 3C). To evaluate whether NF-κB activation is attributed to excessive NOTCH activity, we then treated CADASIL VSMCs with the NOTCH pathway inhibitor DAPT (GSI-IX) (Li et al., 2009). As expected, the expression of *HES1*, a typical target gene of NOTCH3, was inhibited by DAPT (Fig. S1F). In addition, DAPT treatment exerted inhibitory effects on NF-κB target genes, similar to those produced by the NF-κB inhibitor caffeic acid phenethyl ester (CAPE) treatment (Natarajan et al., 1996) (Fig. 3D). These data indicated that upregulation of the NOTCH pathway genes at least partially contributed to NF-κB activation in CADASIL VSMCs.

### CADASIL VSMCs exhibited hyperproliferation and abnormal cytoskeleton structure

**Figure 4:**
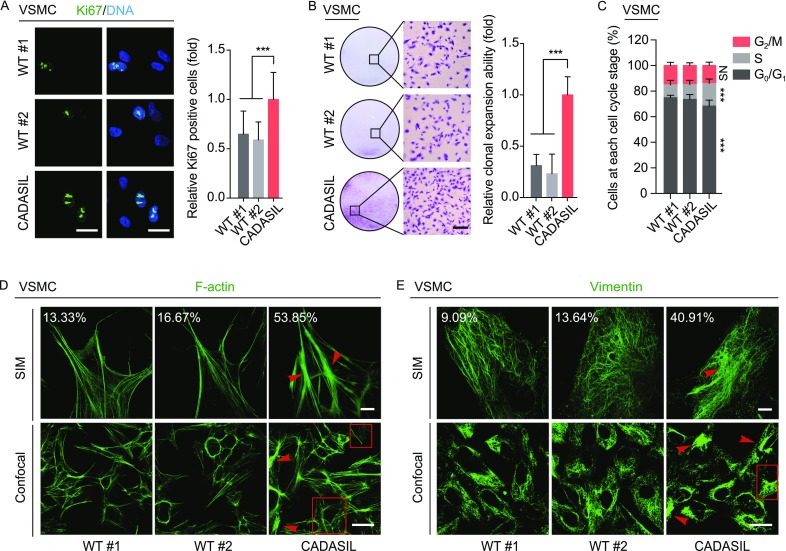
CADASIL VSMCs exhibited hyperproliferation and abnormal cytoskeleton structure. (A) Immunofluorescence staining of Ki67 in WT and CADASIL VSMCs. Nuclei were stained with Hoechst 33342. Scale bar, 25 μm. The relative percentages of Ki67-positive cells (CADASIL was taken as reference) are shown to the right. Data are presented as mean ± SD, *n* = 8. ***P < 0.001. (B) Clonal expansion analysis of WT and CADASIL VSMCs. Representative images of crystal violet staining are shown to the left, Scale bar, 100 μm. The statistical analyses of relative clonal expansion abilities are shown to the right (CADASIL was taken as reference). Data are shown as mean ± SD, *n* = 3. ***P < 0.001. (C) Cell cycle analysis of WT and CADASIL VSMCs. Data are shown as mean ± SD, *n* = 3. ***P < 0.001; NS, not significant. (D) 3D-SIM (top) and confocal microscope images (bottom) of F-actin showing increased aggregation of parallel microfilaments and scattered nodes (arrow heads) in CADASIL VSMCs. Inside the red rectangle is a substantially normal cell. Scale bar of 3D-SIM images, 5 μm. Scale bar of confocal microscope images, 50 μm. The percentages of cells with abnormal F-actin in SIM images are shown. (E) 3D-SIM (top) and confocal microscope images (bottom) showing increased percentage of cells with aggregated vimentin (arrow heads) in CADASIL VSMCs. Inside the red rectangle is a substantially normal cell. Scale bar of 3D-SIM images, 5 μm. Scale bar of confocal microscope images, 25 μm. The percentages of cells with abnormal vimentin in SIM images are shown

Consistent with the aforementioned results, Ki67 immunofluorescence and clonal expansion assays showed that CADASIL VSMCs exhibited greater proliferation ability than WT VSMCs (Fig. 4A and 4B). Cell cycle analysis revealed a higher proportion of S-phase cells for CADASIL VSMCs than for WT VSMCs (Fig. 4C). It has been shown that abnormal VSMC migration may contribute to vasculature disorder. Accordingly, we examined the migration abilities of VSMCs and found no changes in CADASIL VSMCs (Fig. S1G). Given that transcriptomic data suggested that cytoskeleton structures were dysregulated in CADASIL VSMCs, a possibility consistent with previous reports (Domenga et al., 2004; Tikka et al., 2012), we further investigated cytoskeleton changes via immunofluorescence analysis. Compared with WT VSMCs, CADASIL VSMCs had more parallel microfilaments aggregated into robust bundles and distributed as scattered nodes in the cytosol (arrow heads) (53.85% of CADASIL VSMCs compared with 13.33% and 16.67% of cells in the two WT VSMC lines) (Fig. 4D) (Domenga et al., 2004; Tikka et al., 2012). Vimentin was also prone to form a dense bundle-like architecture in CADASIL VSMCs (arrow heads) (40.91% of CADASIL VSMCs relative to 9.09% and 13.64% of cells of the two WT VSMC lines) (Fig. 4E). No abnormalities were observed in the structures of microtubule and vinculin (an adhesion junction component) in CADASIL VSMCs (Fig. S1H). Collectively, our data suggested that CADASIL VSMCs had increased proliferative ability and an abnormal cytoskeleton structure.

### Transcriptional changes associated with NOTCH and NF-κB signaling pathway, or the cytoskeleton in CADASIL VSMCs were not detectable in CADASIL VECs

**Figure 5:**
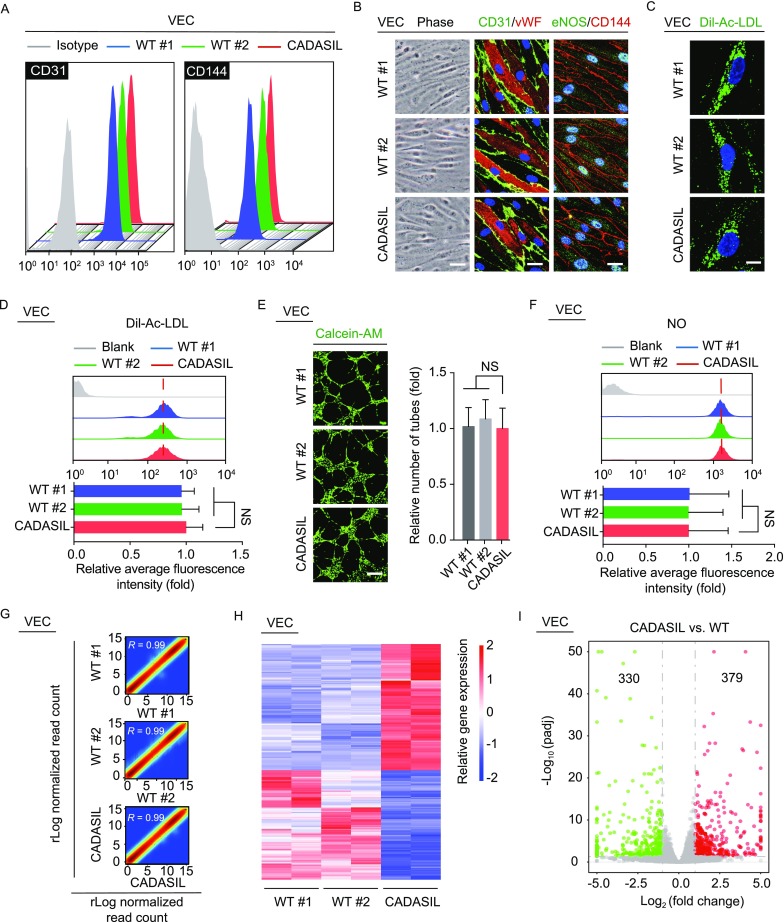

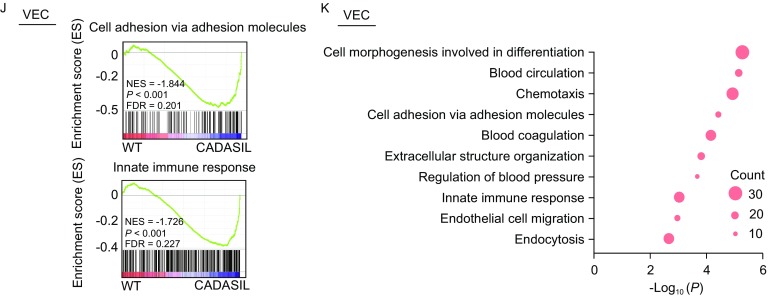
Transcriptional profiling of CADASIL VECs. (A) Flow cytometry analysis of VEC-specific markers CD31 and CD144 in WT and CADASIL VECs. (B) Phase-contrast images of VECs are shown to the left. Scale bar, 50 μm. Immunofluorescence staining of VEC-specific markers, CD31, vWF, CD144 and eNOS, are shown to the right. Nuclei were stained with Hoechst 33342. Scale bar, 25 μm. (C) Immunofluorescence staining of Dil-Ac-LDL in WT and CADASIL VECs. Nuclei were stained with Hoechst 33342. Scale bar, 10 μm. (D) Flow cytometry analysis of Dil-Ac-LDL uptake abilities in WT and CADASIL VECs. The relative average fluorescence intensities are shown in the bottom (CADASIL was taken as reference). Data are presented as mean ± SD, *n* = 3. NS, not significant. (E) The abilities of *in vitro* tube formation in WT and CADASIL VECs. Scale bar, 100 μm. The relative numbers of tubes are shown to the right (CADASIL was taken as reference). Data are presented as mean ± SD, *n* = 3. NS, not significant. (F) Flow cytometry analysis of nitric oxide (NO) levels in WT and CADASIL VECs. The relative average fluorescence intensities are shown in the bottom (CADASIL was taken as reference). Data are presented as mean ± SD, *n* = 3. NS, not significant. (G) Scatter plots showing the correlation between replicates of WT and CADASIL VECs. (H) Heatmap illustrating differentially expressed genes in WT and CADASIL VECs. (I) Volcano plot showing the number of upregulated (red dot) and downregulated (green dot) genes in CADASIL VECs. (J) Gene set enrichment analysis (GSEA) plots showing representative GO-BP terms enriched in CADASIL VECs. (K) GO enrichment analysis of upregulated genes in CADASIL VECs

CADASIL-associated phenotypic changes in other types of vascular wall cells, such as VECs, have not yet been characterized. To better understand CADASIL-specific phenotypes in different layers of the vascular wall, we differentiated iPSCs into VECs. CADASIL VECs and WT VECs expressed similar levels of VEC-specific markers (CD31, vWF, CD144 and eNOS) (Fig. 5A and 5B). Canonical functional analyses of VECs, including acetylated low density lipoprotein (Dil-Ac-LDL) uptake, *in vitro* tube formation and nitric oxide (NO) synthesis, demonstrated that CADASIL VECs had no obvious functional defects compared with WT control cells (Fig. 5C–F).

We then performed RNA sequencing to determine whether CADASIL VECs had disease-specific transcriptomic changes. The high correlation coefficients between replicates confirmed high reproducibility (Fig. 5G). In total, 379 upregulated genes and 330 downregulated genes were identified in CADASIL VECs (Fig. 5H and 5I), which had fewer differentially expressed genes than CADASIL VSMCs. GSEA and GO-BP analyses revealed that cell-cell adhesion via adhesion molecules and innate immune response were enriched in CADASIL VECs (Fig. 5J and 5K). However, the upregulated genes in CADASIL VSMCs that were associated with the NOTCH signaling pathway, the NF-κB signaling pathway, or the cytoskeleton were not upregulated in CADASIL VECs, suggesting that changes in transcriptional levels of these genes were specific to VSMCs. Upregulated genes in CADASIL VECs were verified by RT-qPCR (Fig. S2A), and downregulated genes in these cells were also verified by GO-BP analysis and RT-qPCR (Fig. S2B and S2C). Taken together, our findings showed that we generated CADASIL-specific VECs, but transcriptional profiling changes associated with the NOTCH signaling pathway, the NF-κB signaling pathway, or the cytoskeleton observed in CADASIL VSMCs were not detected in CADASIL VECs.

### Disease-associated phenotypes observed in CADASIL VSMCs were not detected in CADASIL VECs

**Figure 6:**
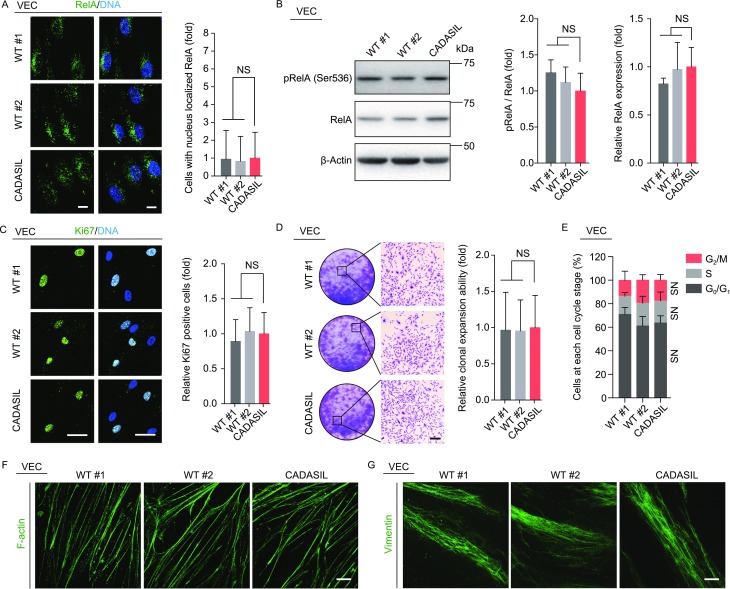
Disease-associated phenotypes observed in CADASIL VSMCs were not detected in CADASIL VECs. (A) Immunofluorescence staining of NF-κB P65 (RelA) in CADASIL VECs. Nuclei were stained with Hoechst 33342. Scale bar, 10 μm. The relative percentages of cells with nucleus localized RelA are shown to the right (CADASIL was taken as reference). Data are presented as mean ± SD, *n* = 3. NS, not significant. (B) Western blot analysis of NF-κB P65 (RelA) and phosphorylated RelA (Ser536) expression levels in WT and CADASIL VECs. β-Actin was used as the loading control. Data are presented as mean ± SD, *n* = 4. NS, not significant. (C) Immunofluorescence staining of Ki67 in WT and CADASIL VECs. Nuclei were stained with Hoechst 33342. Scale bar, 25 μm. The relative percentages of Ki67-positive cells are shown to the right (CADASIL was taken as reference). Data are presented as mean ± SD, *n* = 4. NS, not significant. (D) Clonal expansion analysis of WT and CADASIL VECs. Representative images of crystal violet staining are shown to the left, Scale bar, 100 μm. The statistical analyses of relative clonal expansion abilities are shown to the right (CADASIL was taken as reference). Data are presented as mean ± SD, *n* = 3. NS, not significant. (E) Cell cycle analysis of WT and CADASIL VECs. Data are presented as mean ± SD, *n* = 3. NS, not significant. (F) 3D-SIM images of F-actin in WT and CADASIL VECs. Scale bar, 5 μm. (G) 3D-SIM images of vimentin in WT and CADASIL VECs. Scale bar, 5 μm

To examine whether the phenotypes of CADASIL VSMCs were cell type specific, we next assessed NF-κB activity, cell proliferation ability, and cytoskeleton organization in VECs. Immunofluorescence staining showed that the proportion of cells with nucleus-localized RelA in CADASIL VECs was similar to that in WT VECs (Fig. 6A). Consistently, the expression levels of the phosphorylated RelA (pRelA) were similar between CADASIL VECs and WT VECs (Fig. 6B). Thus, the NF-κB pathway was not activated in CADASIL VECs. Proliferation ability, vimentin and microfilament structures were also normal in CADASIL VECs (Fig. 6C-6G). In addition, no abnormalities in the structures of microtubule, vinculin or tight junction components (ZOI and ClaudinV) were found in CADASIL VECs (Fig. S2D and S2E). Thus, none of the disease-associated phenotypes characterized in CADASIL VSMCs were detected in CADASIL VECs.

### CADASIL VSMCs and VECs were more sensitive to inflammatory stimuli

**Figure 7:**
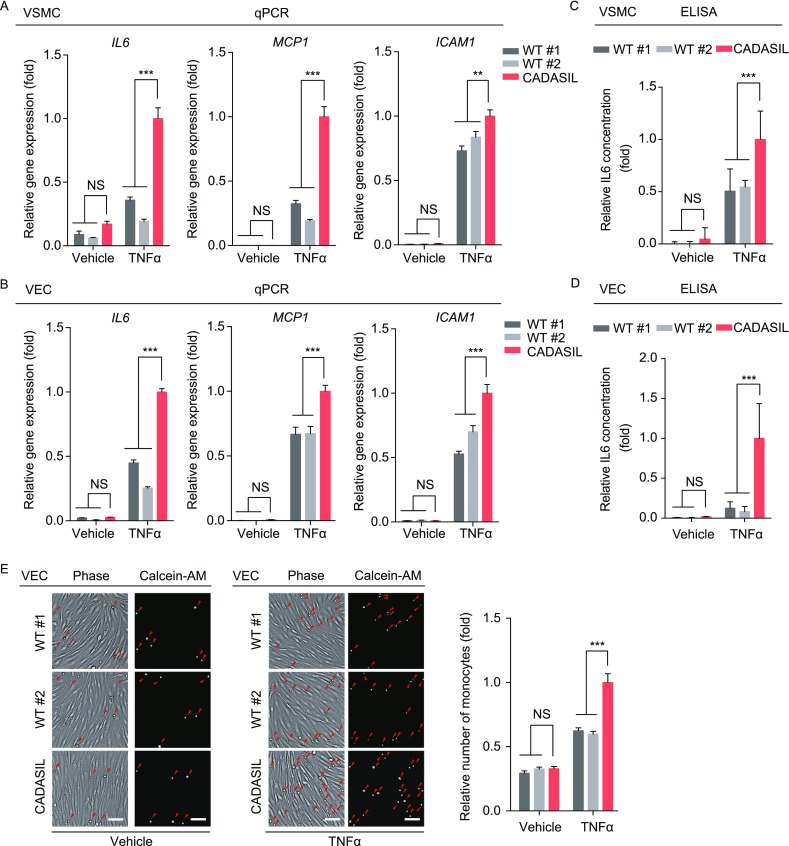
CADASIL VSMCs and VECs were more sensitive to inflammatory stimuli. (A) RT-qPCR analysis showing the expression levels of NF-κB downstream genes, *IL6*, *MCP1*, *ICAM1*, in WT and CADASIL VSMCs under basal and TNFα-induced inflammatory conditions. CADASIL treated with TNFα was taken as reference. Cells were treated with or without 10 ng/mL TNFα for 12 h. Data are shown as mean ± SEM, *n* = 4. ***P < 0.001; **P < 0.01; NS, not significant. (B) RT-qPCR analysis showing the expression levels of NF-κB downstream genes, *IL6*, *MCP1*, *ICAM1*, in WT and CADASIL VECs under basal and TNFα-induced inflammatory conditions. CADASIL treated with TNFα was taken as reference. Cells were treated with or without 10 ng/mL TNFα for 12 h. Data are shown as mean ± SEM, *n* = 4. ***P < 0.001; NS, not significant. (C) ELISA assay showing concentration of IL6 in the culture medium of WT and CADASIL VSMCs under basal and 10 ng/mL TNFα-induced inflammatory conditions. The relative concentration of IL6 is shown (CADASIL treated with TNFα was taken as reference). Data are shown as mean ± SD, *n* = 3. ***P < 0.001; NS, not significant. (D) ELISA assay showing concentration of IL6 in the culture medium of WT and CADASIL VECs under basal and 10 ng/mL TNFα-induced inflammatory conditions. The relative concentration of IL6 is shown (CADASIL treated with TNFα was taken as reference). Data are shown as mean ± SD, *n* = 3. ***P < 0.001; NS, not significant. (E) Monocyte adhesion to WT and CADASIL VECs under basal and 10 ng/mL TNFα-induced inflammatory conditions. Red arrow heads indicate monocytes. Scale bar, 50 μm. The relative numbers of adhered monocytes are shown to the right (CADASIL treated with TNFα was taken as reference). Data are shown as mean ± SD, *n* = 3. ***P < 0.001; NS, not significant

Blood vessels are readily exposed to various endogenous or exogenous inflammatory stimuli (Wang et al., 2018a). Accordingly, we analyzed the expression levels of cytokines, chemokines and adhesion molecules in VSMCs and VECs under TNFα-induced inflammatory condition. Upon stimulation, the expression levels of NF-κB downstream genes, *IL6*, *MCP1*, *ICAM1*, were upregulated both in CADASIL VSMCs and VECs compared with those in WT VSMCs and VECs (Fig. 7A and 7B). ELISA assay further confirmed the upregulation of IL6 protein in the culture medium of CADASIL VSMCs and VECs under TNFα-induced inflammatory condition (Fig. 7C and 7D). In addition, we found enhanced monocytes adhesion to CADASIL VECs under TNFα-induced inflammatory condition (Fig. 7E). Altogether, CADASIL VSMCs and VECs demonstrated higher sensitivity to inflammatory stimuli.

## Discussion

**Figure 8:**
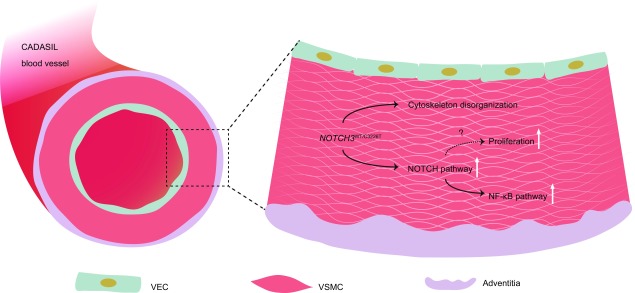
Schematic drawing of the major cellular phenotypes observed in CADASIL VSMCs. The heterozygous *NOTCH3* mutation (c.3226C>T) of VSMCs resulted in increased proliferation ability, cytoskeleton disorganization, activation of NOTCH pathway and NF-κB pathway. However, these disease-associated phenotypes found in CADASIL VSMCs were not observed in CADASIL VECs

In this study, we established an iPSC-based disease model for CADASIL and generated the major cellular components of the vascular media (VSMCs) and intima (VECs), thereby providing a faithful platform for pathogenesis research and drug screening. Using this iPSC disease model, we revealed that increased proliferation ability and abnormal cytoskeleton structures were characteristic features of CADASIL VSMCs (Fig. 8). In addition, we reported that the activation of NF-κB in CADASIL VSMCs was partly attributed to constitutive activation of NOTCH signaling, suggesting a new target for drug discovery.

Increased inflammation and vessel wall remodeling have been widely reported in diverse angiopathies, such as atherosclerosis, diabetes angiopathy, and hypertension angiopathy (Brand et al., 1996; Lontchi-Yimagou et al., 2013; Dinh et al., 2014; Viola and Soehnlein, 2015). In addition, vessel wall remodeling, as manifested by extracellular matrix protein deposition in the VSMC basement membrane and on vessel walls, has been implicated in CADASIL (Dong et al., 2012; Monet-Lepretre et al., 2013; Zhang et al., 2015b; Capone et al., 2016). This possibility is consistent with the upregulation of genes encoding various collagens in our CADASIL VSMCs, which was demonstrated via RNA sequencing analysis. The NF-κB pathway plays an important role in the inflammatory response. Upon stimulation, activated NF-κB translocates into the nucleus to promote the expression of genes encoding cytokines, chemokines and adhesion molecules as well as genes involved in extracellular matrix remodeling. Upregulation of these genes leads to increased vascular inflammation and vascular wall reconstruction, eventually resulting in vascular dysfunction (Baker et al., 2011; Killeen et al., 2014; Wang et al., 2018a). However, it remains unclear whether the NF-κB pathway contributes to CADASIL angiopathy. In our study, transcriptomic data showed that NF-κB target genes associated with vascular inflammation and vessel wall remodeling, including *THBS1*, *MMP1*, *ADAM19* and *TNFSF15* (Bonnefoy et al., 2008; Edwards et al., 2008; Kim et al., 2008; Bin et al., 2009; Penn et al., 2014), were upregulated in CADASIL-specific VSMCs. Among these genes, *THBS1* encodes thrombospondin-1 (TSP1), which has been implicated in vascular dysfunction in pulmonary hypertension and various cerebrovascular diseases (Krishna and Golledge, 2013; Rogers et al., 2017), and *MMP1* (which encodes a matrix metalloprotease) has been shown to promote the occurrence of hypertension, atherosclerosis and thrombosis (Dollery and Libby, 2006; Trivedi et al., 2009; Agrinier et al., 2013). Therefore, the upregulation of NF-κB target genes in CADASIL VSMCs may contribute to CADASIL-associated vascular dysfunction due to vessel wall remodeling and vascular inflammation. In addition, we found a more significant up-regulation of downstream genes of NF-κB in CADASIL VSMCs and VECs under TNFα-induced inflammatory condition. The observed excessive sensitivity to inflammatory stimuli of the CADASIL VSMCs and VECs suggests that CADASIL patients may suffer from cerebral blood vessels overly susceptible to injury under inflammatory conditions, aggravating the progression of angiopathy.

Our study indicates that the R1076C (c.3226C>T) *NOTCH3* mutation may be linked to the activation of NOTCH signaling, at least in human VSMCs. This particular mutation is located within the exon 20 encoding the 27-28 EGFr domain of NOTCH3 (Joutel et al., 1997; Rutten et al., 2016), leading to the addition of a seventh cysteine residue as seen in many typical CADASIL mutations. Upregulation of NOTCH pathway was often associated with cysteine-related pathogenic mutations (Donahue and Kosik, 2004; Haritunians et al., 2005; Baron-Menguy et al., 2017), but how these mutations mediate the activation of NOTCH pathway and occurrence of CADASIL angiopathy remain unclear. One possible explanation is that these mutations interrupt the pairing of the disulfide bond in EGFr domain of NOTCH3 (Rutten et al., 2016). Subsequently, the unpaired cysteine residue causes the self-aggregation of the mutant protein, which inhibits the clearance of NOTCH3 and may enhance NOTCH pathway (Takahashi et al., 2010; Duering et al., 2011; Meng et al., 2012). The NOTCH pathway is evolutionarily conserved and implicated in the regulation of embryonic and organism development in many cell types at various stages (Penton et al., 2012; Andersson and Lendahl, 2014b; Bray, 2016; Siebel and Lendahl, 2017). Canonically, after binding to ligands, the NOTCH intracellular domain (NICD) translocates into the nucleus to promote the expression of downstream genes, mainly hairy and enhancer of split (*HES*) and *HES*-related with YRPW motif (*HEY*) genes. Moreover, a non-canonical branch of NOTCH signaling involves interaction with other signaling pathways, including the NF-κB pathway (Kopan and Ilagan, 2009; Andersson et al., 2011; Andersen et al., 2012; Guruharsha et al., 2012; Andersson and Lendahl, 2014a; Ayaz and Osborne, 2014). Multiple studies have shown that activation of the NOTCH pathway upregulates the NF-κB pathway (Vacca et al., 2006; Shin et al., 2014; Ruan et al., 2016), partially due to interaction between the NICD and NF-κB that prolongs retention of NF-κB in the nucleus (Shin et al., 2006). Consistent with the prior findings, we found that the activation of NF-κB pathway in CADASIL VSMCs was associated with the upregulation of NOTCH signaling and that the NOTCH pathway inhibitor DAPT partially alleviated the expression of NF-κB target genes, providing a new mechanism of and a potential therapeutic target for CADASIL angiopathy.

The causal relationship between NOTCH activation and VSMC proliferation has been implicated in multiple studies (Sweeney et al., 2004; Song et al., 2015; Wu et al., 2016), and abnormal VSMC proliferation is frequently related to and a contributor to pathological processes such as neointima formation and vascular remodeling (Rudijanto, 2007; Chistiakov et al., 2015; Lyon et al., 2016). In our study, both NOTCH activation and increased proliferation ability were observed in CADASIL VSMCs; thus, NOTCH activation may be a factor that contributes to CADASIL angiopathy.

In CADASIL patients and mice, cerebral vessels exhibit reduced response to intraluminal pressure changes and drug stimulation (Chabriat et al., 2000; Pfefferkorn et al., 2001; Joutel et al., 2010; Moreton et al., 2017). In our study, we found abnormalities in the structure of vimentin, a major component of intermediate filaments, in CADASIL VSMCs. NF-κB has been found to positively regulate transcriptional levels of vimentin (Chen et al., 1996; Xu et al., 2016). Accordingly, changes in vimentin structure in our CADASIL VSMCs may be induced by NF-κB activation. We also found abnormal microfilament structure in CADASIL VSMCs, which was consistent with findings from previous studies of CADASIL mice and primary VSMCs from patients (Domenga et al., 2004; Tikka et al., 2012). The NOTCH pathway has been reported to promote the expression of smooth muscle α-actin and the activity of Rho kinase (Noseda et al., 2006; Venkatesh et al., 2011), which may contribute to microfilament disorganization, thereby affecting dynamic regulation of the actin filament structure. In vascular cells, the cytoskeleton not only plays an important role in maintaining tissue and cell morphology and function (Fletcher and Mullins, 2010; Kassianidou and Kumar, 2015) but also helps to sense changes in luminal blood flow and assists with vasoconstriction and vasorelaxation (Henrion et al., 1997; Yamin and Morgan, 2012). We therefore speculated that the abnormal cytoskeleton structure in CADASIL VSMCs may contribute to disease-associated aberrant vasomotor function.

We established CADASIL VECs for the first time, permitting the study of cell type-specific effects of CADASIL in VSMCs and VECs in parallel. Almost none of the changes in cellular phenotypes or gene expression profile observed in CADASIL VSMCs manifested in our CADASIL VECs, suggesting that the relevant *NOTCH3* gene mutation (c.3226C>T, p.R1076C) produces a cell type-specific effect. Notably, VEC abnormalities have been reported in the skin tissues of CADASIL patients, and electron microscopic observations of such tissues have revealed intracytoplasmic vacuoles, cell shrinkage, and extracellular collagen deposition (Ruchoux et al., 1994; Ruchoux and Maurage, 1998). However, it was unclear whether these changes reflect primary abnormalities of VECs or are secondary to vessel wall structural damage or aberrant cell-cell communication. Our transcriptome data reveal some abnormalities in CADASIL VECs, such as changes in innate immunity and cell-cell adhesion. We also observed the upregulation of downstream genes of NF-κB in CADASIL VECs under TNFα-induced inflammatory condition, suggesting that the VECs may contribute to CADASIL angiopathy in the presence of external inflammatory stimuli. It is worth noting that the transcriptomic profiles of *in vitro* cells might also be partially affected by changes in the microenvironment. Thus, the detailed cellular phenotypes and underlying mechanisms awaits further exploration.

Vessel wall is comprised of three layers. Except for the VECs in the intima and the VSMCs in the media, fibroblasts and mesenchymal stem cells (MSCs) in the adventitia also play an important role in vascular-associated diseases (Swift and Weinstein, 2009; Krings et al., 2011; Wang et al., 2018a). However, the role of NOTCH pathway in perivascular MSCs remains unclear. Our preliminary data suggest that CADASIL iPSC-derived MSCs may undergo premature aging (data not shown), which may contribute to depletion of perivascular MSCs in CADASIL. Thus the abnormalities of CADASIL MSCs likely reflect the previously reported changes of pericytes in CADASIL (Gu et al., 2012; Craggs et al., 2015; Ghosh et al., 2015).

CADASIL being a rare disease, only one patient was involved in our study. Despite that we used two WTs, isogenic disease-free control line may be even more helpful to establish in the future for the better simulation of disease phenotypes and understanding of underlying mechanisms (Li et al., 2011; Liu et al., 2011b; Wang et al., 2017). At the same time, many studies have pointed out that gene-corrected isogenic iPSCs have a good application prospect in the treatment of many diseases, such as the repair of damaged cardiovascular systems using iPSC-derived vascular cells (Ye et al., 2014; Jung et al., 2017; Zhang et al., 2018a). Therefore, generation of gene-corrected isogenic iPSCs may also help searching therapeutic maneuvers for CADASIL.

In summary, we have modeled CADASIL-related vascular pathologies using an iPSC-based disease model and generated corresponding VSMCs and VECs for phenotypic and mechanistic studies. Our study not only unearthed novel disease-associated cellular phenotypes and gene expression changes, thereby generating clues for future pathogenesis research, but also provided potential therapeutic strategies for CADASIL.

## Materials and methods

### Cell culture

Human fibroblasts were cultured in high glucose DMEM (Invitrogen) supplemented with 10% FBS (Gemcell), 0.1 mmol/L non-essential amino acids (NEAA, Gibco), 2 mmol/L GlutaMAX (Gibco) and 1% penicillin/streptomycin (Invitrogen). Human iPSCs were maintained on mitomycin C-inactivated mouse embryonic fibroblast (MEF) feeder in human ESC culture medium containing 80% DMEM/F12 (Gibco), 20% Knockout Serum Replacement (Gibco), 0.1 mmol/L NEAA (Gibco), 2 mmol/L GlutaMAX (Gibco), 55 μmol/L β-mercaptoethanol (Invitrogen) and 10 ng/mL FGF2 (Joint Protein Central) or on Matrigel (BD Biosciences) in mTeSR medium (STEMCELL Technologies). Human VSMCs were cultured in N2B27 medium (Wang et al., 2018a) supplemented with 10 ng/mL PDGF-BB (Peprotech). Human VECs were cultured in EGM-2 medium (Lonza).

### Non-integrative iPSCs generation

Fibroblasts were cultured from the skin of a CADASIL patient harboring a heterozygous *NOTCH3* mutation (c.3226C>T, p.R1076C) and two healthy controls. Primers for identification of the heterozygous *NOTCH3* mutation: *NOTCH3* forward primer, CACGTACCTCCTGCTAGTGTGAGCCGAA, *NOTCH3* reverse primer, AGGCTGAAGCAGAAGAATCACCTGAACCC. The study was approved by the ethics committee of the Peking University First Hospital and a written informed consent was obtained. iPSCs were generated by electroporating fibroblasts with episomal vectors including pCXLE-hOCT3/4-shp53-F, pCXLE-hSK and pCXLE-hUL as previously described (Li et al., 2011; Okita et al., 2011; Liu et al., 2014; Fu et al., 2016; Wang et al., 2017). The generated iPSCs were manually picked and maintained on MEF feeder.

### RT-PCR and RT-qPCR

Total RNA extraction and cDNA synthesis were performed using TRIzol reagent (Invitrogen) and GoScript Reverse Transcription System (Promega). For RT-PCR, cDNA was applied to PCR using primers including: *GAPDH* forward primer, TCGGAGTCAACGGATTTGGT, *GAPDH* reverse primer, TTGCCATGGGTGGAATCATA. *NANOG* forward primer, ACAACTGGCCGAAGAATAGCA, *NANOG* reverse primer, GGTTCCCAGTCGGGTTCAC. *OCT4* forward primer, GGGTTTTTGGGATTAAGTTCTTCA, *OCT4* reverse primer, GCCCCCACCCTTTGTGTT. *SOX2* forward primer, CAAAAATGGCCATGCAGGTT, *SOX2* reverse primer, AGTTGGGATCGAACAAAAGCTATT. RT-qPCR was carried out with SYBR Green PCR Master Mix (Bio-Rad) on a CFX-384 RT-qPCR system (Bio-Rad). The relative expression of genes was normalized by *18S *rRNA transcript. Primers used for RT-qPCR are listed in Table S1.

### Teratoma analysis

Briefly, 5 × 10^6^ iPSCs per line were injected subcutaneously into NOD/SCID mice (male, 6–8 weeks). About three months after injection mice were killed and teratomas were accessed for immunofluorescence. All murine experiments were conducted with the approval by the Institute of Biophysics, Chinese Academy of Science.

### Bisulfite sequencing of the *OCT4* promoter

Genomic DNA was extracted with Qiagen Blood and Tissue kit. Bisulfite modification of genomic DNA was carried out using CpGenome Fast DNA Modification Kit (Millipore) following the manufacturer’s instructions. The modified genomic fragment of *OCT4* promoter was amplified using LA Taq Hot StartVersion (TAKARA) as previously described (Duan et al., 2015). The PCR products were then purified using PCR purification kit (Qiagen) and subsequently cloned into the pMD20 T vector (TAKARA). Seven clones from each sample were sequenced. Primers used for PCR: me-*OCT4* forward primer, ATTTGTTTTTTGGGTAGTTAAAGGT, me-*OCT4* reverse primer, CCAACTATCTTCATCTTAATAACATCC.

### Cell cycle analysis

For cell cycle analysis, 1 × 10^6^ cells were collected and fixed in 75% ice-cold ethanol at −20 °C overnight. The cells were then washed twice with PBS and stained with 0.02 mg/mL propidium iodide and 0.2 mg/mL RNase at 37 °C for 30 min. Cells were examined using a flow cytometry (BD LSRFortesa) and cell-cycle phase distributions were analyzed by ModFit software (Wang et al., 2018b).

### Clonal expansion assay

The single-cell clonal expansion assay was carried out as previously described (Wu et al., 2018). Briefly, 2,000 cells were seeded onto 12-well plate and each line was analyzed in triplicate. The cell density was analyzed by ImageJ2x 669 2.1.4.7 software after crystal violet staining.

### Generation of VSMCs

VSMC differentiation was carried out as previously described (Patsch et al., 2015). Briefly, iPSCs were unicellularized using TrypLE Express (Gibco) and seeded onto matrigel-coated 6-well plates at a density of 3 × 10^5^ per well. After incubated in mTeSR supplemented with 10 µmol/L Y-27632 (Selleck) for one day, cells were then cultured in the N2B27 medium supplemented with 25 ng/mL BMP4 (R&D) and 8 μmol/L CHIR99021 (Selleck) for 3 days. Finally, cells were cultured in N2B27 medium supplemented with 10 ng/mL PDGF-BB (Peprotech) and 2 ng/mL Activin A (HumanZyme) for another 2 days and sorted after stained with anti-human CD140b-PE (BD biosciences, 558821, 1:200) by a flow cytometry (BD FACSAria IIIu). IgG-PE (BD biosciences, 555749) was used as an isotype control.

### Generation of VECs

The iPSC clones were picked onto matrigel-coated 6-well plates in mTeSR medium. The next day culture medium was changed to EGM-2 medium (Lonza) supplemented with 25 ng/mL BMP4 (R&D), 3 µmol/L CHIR99021 (Selleck), 3 µmol/L IWP2 (Selleck) and 4 ng/mL FGF2 (Joint Protein Central) for 3 days. The cells were then cultured in EGM-2 medium supplemented with 50 ng/mL VEGF (HumanZyme), 10 ng/mL IL6 (Peprotech) and 20 ng/mL FGF2 (Joint Protein Central) for another 3 days. VECs were stained with anti-human CD34-FITC (BD biosciences, 555821, 1:200), anti-human CD201-PE (BD Biosciences, 557950, 1:200), and anti-human CD144-APC (BD Biosciences 561567, 1:200) and then sorted by a flow cytometry (BD FACSAria IIIu). IgG-FITC (BD biosciences, 555748), IgG-PE (BD biosciences, 555749) and IgG-APC (BD Biosciences, 555751) were used as isotype controls (Yang et al., 2017).

### Identification of VEC surface markers

5 × 10^5^ cells were collected and stained with anti-human CD31-FITC (BD biosciences, 557508, 1:100) and anti-human CD144-PE (BD Biosciences 561714, 1:200) and analyzed by a flow cytometry (BD FACSAria IIIu). IgG-FITC (BD biosciences, 555748) and IgG-PE (BD biosciences, 555749) were used as isotype controls.

### Measurement of nitric oxide (NO)

About 5 × 10^5^ VECs were treated with DAF-FM (Molecular Probes) to detect intracellular NO according to the manufacturer’s instructions. After stained for 30 min at room temperature, cells were quantified by a flow cytometry (BD FACSAria IIIu). The average fluorescence intensities were analyzed by FlowJo_V10 software.

### Dil-Ac-LDL uptake assay

In brief, ECs were incubated with Dil-Ac-LDL (Molecular Probes) in EC culture medium for 6 h. For FACS analysis, cells were collected and analyzed by a flow cytometry (BD FACSAria IIIu). The average fluorescence intensities were analyzed by FlowJo_V10 software.

### *In vitro* tube formation assay

Briefly, 6.5 × 10^4^ VECs were suspended in 500 μL medium and then seeded on matrigel-coated 24 well plates. After cells were incubated for 8 h at 37 °C, formed tube-like structures were stained with Calcein-AM (Invitrogen) and visualized by fluorescence microscope (Olympus).

### Transwell migration assay

Briefly, 2 × 10^4^ cells were seeded on top of the 0.8 μm filters of Boyden chambers (Millipore) in serum deprivation medium. Then the filters were placed into 24 culture plate wells containing normal culture medium. Cells were allowed to migrate for 24 h in a humidified incubator at 37 °C, and 3 replicates were performed for each line. After incubation, the filter inserts were fixed with 4% paraformaldehyde and then the migrated cells were stained by purple crystal 30 min at room temperature. After washing 3 times using PBS, photograph was taken using light microscope and the number of migrated cells was counted with ImageJ2x 669 2.1.4.7.

### Monocyte adhesion assay

Monocyte adhesion assay was carried out as previously described (Wang et al., 2018a). Briefly, 2 × 10^5^ VECs were seeded in each well of 12-well plate. The next day, VECs were treated with or without 10 ng/mL TNFα (Peprotech) for 12 h. Then, 2 × 10^6^ monocytes were co-cultured with VECs for 2 h after stained with Calcein-AM (Invitrogen). The monocytes adhered on endothelium were visualized by fluorescence microscope (Olympus) after rinsed by PBS for 5 times carefully. The number of adhered monocytes was analyzed by ImageJ2x 669 2.1.4.7 software.

### IL6 ELISA

Cells cultured in equal volume of medium were treated with or without 10 ng/mL TNFα (Peprotech) for 12 h. The cell culture medium was collected and filtered to remove cell debris, and the cell number was calculated at the same time. Concentration of secreted IL6 in the culture medium of cells was detected using Biolegend’s ELISA kit (Cat. No. 430504) following the manufacturer’s instructions, and 4–6 replicates were performed for each sample. The final concentration was obtained by normalization according to the number of cells and was recorded as pg/mL per 10^4^ cells.

### Western blott

About 1 × 10^6^ cells were lysed with 100 μL RIPA buffer [50 mmol/L Tris-HCl (pH = 7.5), 150 mmol/L NaCl, 1% NP-40, 0.5% sodium deoxycholate, 0.1% SDS] supplemented with NaF, NaVO_4_ and a protease-inhibitor mixture (Roche). Typically 20 µg of protein were separated by SDS-PAGE and then transferred to a PVDF membrane (Millipore). After blocked with 5% skimmed milk powder, the membrane was incubated with primary antibody overnight at 4 °C and then with HRP-conjugated secondary antibody (1:5000). The quantification of Western blot was performed with Image Lab software for ChemiDoc XRS system (Bio-Rad) and the expression levels of protein were analyzed by ImageJ2x 669 2.1.4.7 software.

Primary antibodies for Western blot include anti-NF-κB P65 (RelA) (CST, 8242S; 1:2,000), anti-phospho-NF-κB P65 (Ser536) (pRelA) (CST, 3033S; 1:1,000), anti-β-Actin (Santa Cruz, sc-69879; 1:5000).

### Immunofluorescence microscopy

Cells seeded on microscope coverslips were fixed with 4% formaldehyde for 20–30 min, permeabilized with 0.4% Triton X-100 in PBS for 10–20 min, and blocked with 10% donkey serum in PBS for 1 h at room temperature. Cells were then incubated with primary antibody (diluted with 1% donkey serum in PBS) overnight at 4 °C and fluorescence-labeled secondary antibody (Invitrogen; 1:500 diluted with 1% donkey serum in PBS) at room temperature for 1 h the next day. Hoechst 33342 (Invitrogen; 1:1,000) was used to stain nuclear DNA.

Primary antibodies for immunofluorescence include anti-NANOG (Abcam, ab21624; 1:100), anti-SOX2 (Santa Cruz, sc-17320; 1:200), anti-OCT4 (Santa Cruz, sc-365509; 1:200), anti-TUJ1 (Sigma, T2200; 1:500), anti-α-SMA (Sigma, A5228; 1:200), anti-FOXA2 (CST, 8186S; 1:200), anti-Vimentin (Abcam, ab8978; 1:250), anti-α-Tubulin (Sigma, T5168; 1:500), anti-Vinculin (Sigma, V9131; 1:100), anti-ZOI (Abcam, ab96587; 1:200), anti-ClaudinV (Abcam, ab15106; 1:200), anti-SM22 (Abcam, ab14106; 1:200), anti-Calponin (Dako, M3556; 1:200), anti-vWF (Dako, A0082; 1:200), anti-eNOS (BD, 610296; 1:100), anti-VE-cadherin (CD144) (CST, 2158S; 1:100), anti-human CD31-FITC (BD biosciences, 557508, 1:100), anti-Ki67 (Vector Laboratories, ZA0731; 1:500), Phalloidin (F-actin) (Invitrogen, A22287; 1:50), anti-NF-κB P65 (RelA) (CST, 8242S; 1:200).

### RNA-seq library construction and data quality control

VSMCs and VECs at passage 2 were collected for RNA-seq analysis using Illumina sequencing platform. RNA sequencing libraries were prepared as previously reported (Geng et al., 2018; Wang et al., 2018a). Briefly, RNA integrity was examined by the Bioanalyzer 2100 system (Agilent Technologies). Sequencing libraries were constructed using NEB Next UltraTM RNA Library Prep Kit for Illumina (NEB) and sequenced on Illumina Hiseq X Ten platform. All of the sequencing reads were cleaned to remove any artificial sequences and reads with more than 10% low-quality bases.

### RNA-seq data processing

RNA-seq data processing was performed as previously described (Zhang et al., 2015a; Geng et al., 2018; Wang et al., 2018a). Sequencing reads were trimmed and mapped to hg19 human genome using hisat2 software (v2.0.4) (Kim et al., 2015). The transcriptional expression level of each gene was counted by HTSeq (v0.6.1) (Anders et al., 2015). Differentially expressed genes (DEGs) were computed using DESeq2 with the threshold of adjusted *P* value (Benjamini-Hochberg) less than 0.05 and |Log_2_(fold change)| more than 1 (Love et al., 2014). The correlation between replicates of each sample was evaluated by the Pearson correlation coefficient (R), which was based on DESeq2 regularized-logarithm (rLog) normalized read count. Gene ontology (GO) and pathway enrichment analysis was conducted by Metascape (http://www.metascape.org/) (Tripathi et al., 2015). Gene set enrichment analysis (GSEA) was performed using GSEA software (Subramanian et al., 2007). Transcription levels of NF-κB target genes were analyzed as below. The NF-κB target genes were identified according to database (Siggers et al., 2015; Li et al., 2017). NF-κB target genes with a *P* value less than 0.01 were taken into consideration and *P* values of Log_2_(Fold change) between NF-κB target genes (*P* < 0.01) and other genes were calculated by Two-sample Kolmogorov-Smirnov test. The RNA-seq data have been deposited to the NCBI Gene Expression Omnibus (GEO) database with accession number GSE124500.

### 3D-SIM super-resolution microscopy and image analysis

After cells were stained, 3D-SIM images of VSMCs and VECs were acquired on the DeltaVision OMX V3 imaging system (GE Healthcare) with a 100×/1.40 NA oil objective (Olympus UPlanSApo), solid-state multimode lasers (488 nm, 405 nm, 561 nm) and electron-multiplying CCD (charge-coupled device) cameras (Evolve 512 × 512, Photometrics). Serial Z-stack sectioning was done at 125 nm intervals for SIM mode. To obtain optimal images, immersion oil with refractive indices of 1.516 was used for cells on glass coverslips. The microscope was routinely calibrated with 100 nm fluorescent spheres to calculate both the lateral and axial limits of image resolution. SIM image stacks were reconstructed using softWoRx 6.1.1 (GE Healthcare) with the following settings: pixel size 39.5 nm; channel-specific optical transfer functions; Wiener filter constant 0.0010; discard Negative Intensities background; drift correction with respect to first angle; custom K0 guess angles for camera positions. The reconstructed images were further processed for maximum-intensity projections with softWoRx 6.1.1. Pixel registration was corrected to be less than 1 pixel for all channels using 100 nm Tetraspeck beads.

For imaging and analysis of cytoskeletal structures, the statement of the normal cytoskeletal structures were based on the morphology of the WTs in the representative picture. The normal cytoskeletal structures are as follows: the microfilaments should without obvious robust bundles or nodular structures (Domenga et al., 2004; Tikka et al., 2012); the intermediate filaments and microtubules should be in the form of a filament-like structure distributed in a network without significant aggregation (Fogl et al., 2016; Fuertes-Alvarez et al., 2018; Tu et al., 2018); the adhesion junction protein vinculin should be in a punctiform structure and co-localizes with the microfilaments near the cell membrane (Tikka et al., 2012); the tight junction proteins are membrane-localized proteins that resemble the pattern of other VEC surface markers such as CD31 and CD144 (Lee et al., 2018; Zhang et al., 2018b).

### Statistical analysis

CADASIL was compared with the mean of the two WTs using unpaired *t*-test. RT-qPCR results were analyzed using one-way ANOVA and Bonferroni Post Hoc test. All results of experiments with TNFα treatment were analyzed by two-way ANOVA and Sidak’s multiple comparisons test. All the analyses were conducted using Graph-Pad Prism Software and *P *value less than 0.05 were considered statistically significant. *P* > 0.05 (NS), *P* < 0.05 (*), *P* < 0.01 (**) and *P* < 0.001 (***).

## Electronic supplementary material

Below is the link to the electronic supplementary material.

Supplementary material 1 (PDF 3388 kb)

Supplementary material 2 (XLSX 14 kb)
