# Supplementary material for: Modeling CADASIL vascular pathologies with patient-derived induced pluripotent stem cells
Source: Protein Cell. 2019 Feb 18;10(4):249–71. doi: 10.1007/s13238-019-0608-1 (PMC6418078; doi:10.1007/s13238-019-0608-1)
Supplement: Supplementary file 1 — Supplementary material 1 (PDF 3388 kb) [file 13238_2019_608_MOESM1_ESM.pdf]

Figure S1. (A) Summary of reprogramming efficiency of WT and CADASIL fibroblasts. (B) RT-qPCR analysis showing little residual episomal vector element *EBNA-1* in WT and CADASIL iPSCs. Human fibroblasts 4 days after electroporated with pCXLE-hOCT3/4-shp53-F, pCXLE-hSK and pCXLE-hUL (fibroblast (+)) were used as positive control; fibroblasts without electroporation (fibroblast (-)) and human ESCs (hESCs) were used as negative control. Data are shown as mean ± SD. n = 4. NS, not significant. (C) Verification of upregulated genes in CADASIL VSMCs by RT-qPCR. CADASIL was taken as reference. Data are presented as mean ± SEM, n = 4. ***$P <$ 0.001. (D) GO enrichment analysis of downregulated genes in CADASIL VSMCs. (E) Verification of downregulated genes in CADASIL VSMCs by RT-qPCR. CADASIL was taken as reference. Data are presented as mean ± SEM, n = 4. ***$P < 0.001$; **$P <$ 0.01. (F) RT-qPCR analysis of *HES1* expression in CADASIL VSMCs after treated with 20 μmol/L DAPT for 18 h. Vehicle was taken as reference. Data are presented as mean ± SEM, n = 4. ***$P < 0.001$. (G) Transwell migration analysis of WT and CADASIL VSMCs. Representative images of crystal violet staining are shown to the left, Scale bar, 50 μm. The relative number of migrated cells are shown to the right (CADASIL was taken as reference). Data are presented as mean ± SD, n = 3. NS, not significant. (H) 3D-SIM images of microtubule structure in CADASIL VSMCs (top). Scale bar, 5 μm. Confocal microscope images of vinculin (one of the adhesion junction components) structure in CADASIL VSMCs (bottom). Scale bar, 10 μm.

Figure S2. (A) Verification of upregulated genes in CADASIL VECs by RT-qPCR. CADASIL was taken as reference. Data are presented as mean ± SEM, n = 4. ***$P <$ 0.001. (B) GO enrichment analysis of downregulated genes in CADASIL VECs. (C) Verification of downregulated genes in CADASIL VECs by RT-qPCR. CADASIL was taken as reference. Data are presented as mean ± SEM, n = 4. ***$P < 0.001$; **$P <$ 0.01; *$P < 0.05$. (D) 3D-SIM images of microtubule structure in CADASIL VECs (top). Scale bar, 5 μm. Confocal microscope images of vinculin structure in CADASIL VECs (bottom). Scale bar, 10 μm. (E) Immunofluorescence staining of ZOI and ClaudinV in WT and CADASIL VECs. Nuclei were stained with Hoechst 33342. Scale bar, 25 μm.

Figure S1

A

Fibroblast	Number of initial cells	Number of iPSC colonies
WT #1	1.5 × 10^6	23
WT #2	1.5 × 10^6	25
CADASIL	1.5 × 10^6	19

B

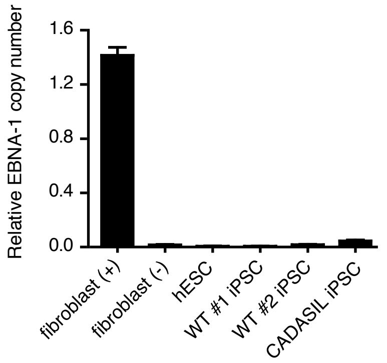

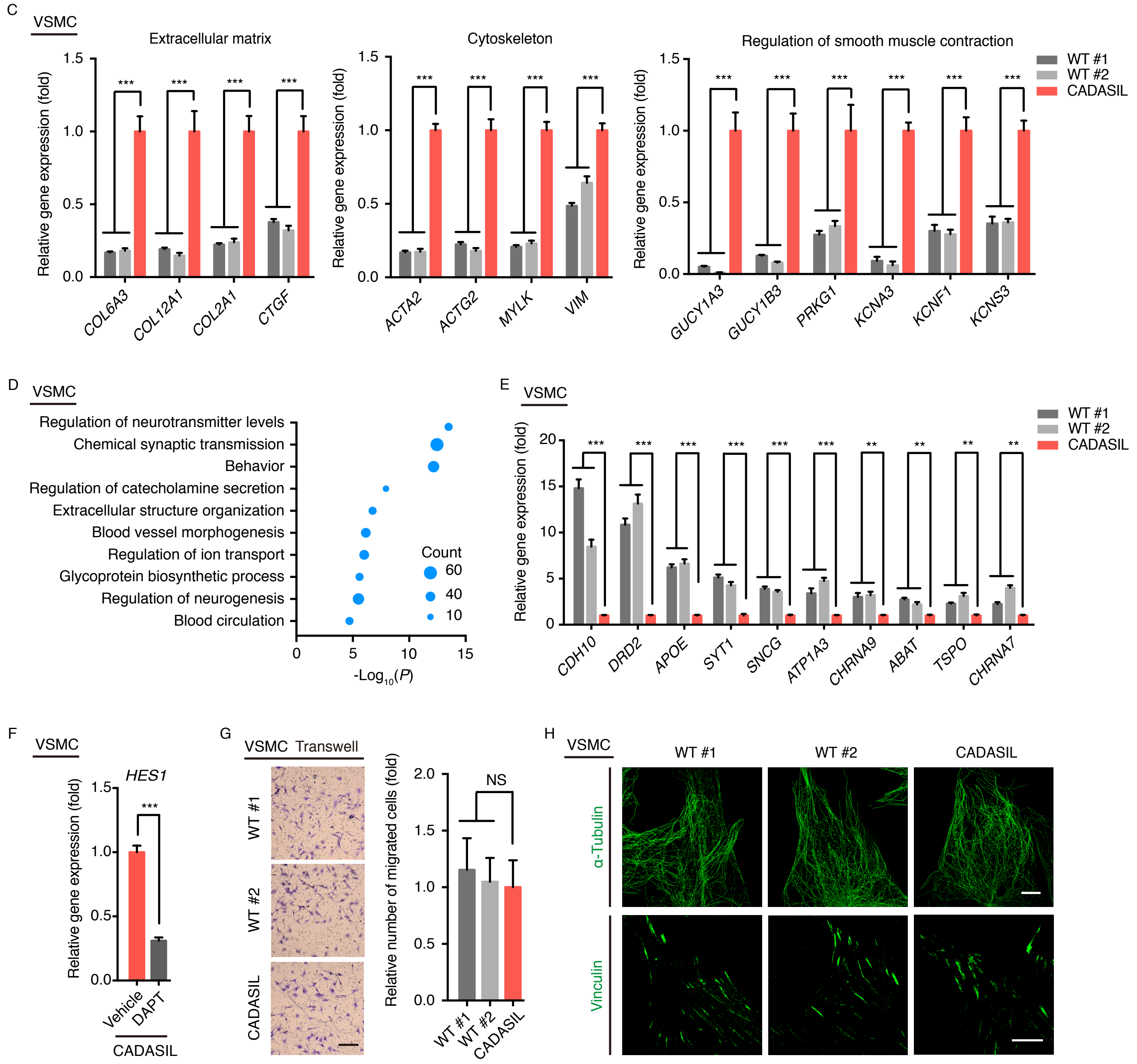

Figure S2

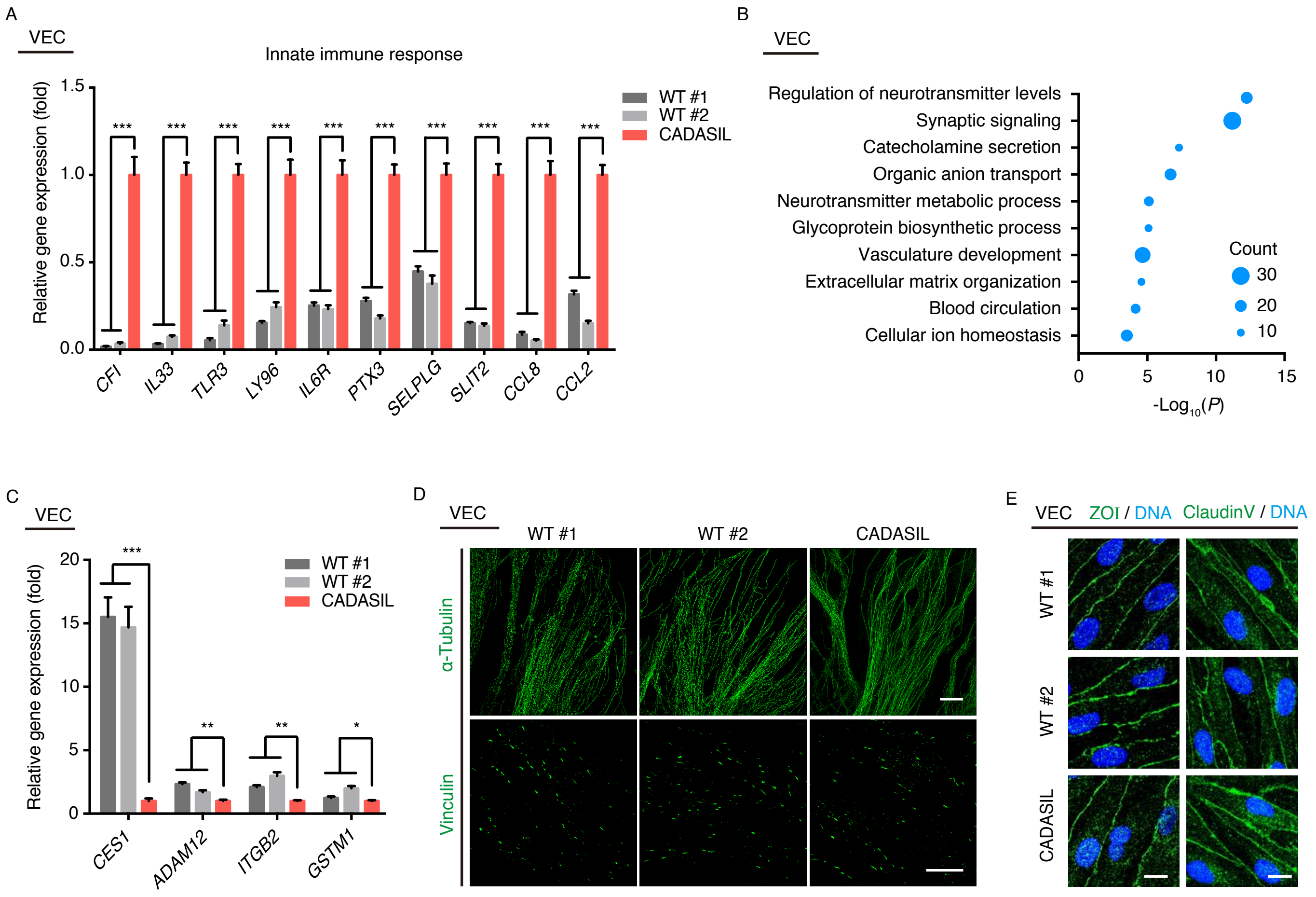

A
VEC
Innate immune response
Relative gene expression (fold)
1.5
1.0
0.5
0.0
***
WT #1
WT #2
CADASIL
CFI
IL33
TLR3
LY96
IL6R
PTX3
SELPLG
SLIT2
CCL8
CCL2
B
VEC
Regulation of neurotransmitter levels
Synaptic signaling
Catecholamine secretion
Organic anion transport
Neurotransmitter metabolic process
Glycoprotein biosynthetic process
Vasculature development
Extracellular matrix organization
Blood circulation
Cellular ion homeostasis
Count
30
20
10
0
5
10
15
-Log10(P)
C
VEC
Relative gene expression (fold)
20
15
10
5
0
***
**
*
WT #1
WT #2
CADASIL
CES1
ADAM12
ITGB2
GSTM1
D
VEC
WT #1
WT #2
CADASIL
α-Tubulin
Vinculin
E
VEC
ZOI / DNA
ClaudinV / DNA
WT #1
WT #2
CADASIL
